# Higher gamma-glutamyl transferase levels are associated with an increased risk of incident systemic sclerosis: a nationwide population-based study

**DOI:** 10.1038/s41598-023-49183-1

**Published:** 2023-12-11

**Authors:** Oh Chan Kwon, Kyungdo Han, Min-Chan Park

**Affiliations:** 1 Division of Rheumatology, Department of Internal Medicine, Yonsei University College of Medicine, Seoul, South Korea; 2 Department of Statistics and Actuarial Science, Soongsil University, 369 Sangdo-ro, Dongjak-gu, Seoul, 06978 South Korea; 3 Gangnam Severance Hospital, Yonsei University College of Medicine, 211 Eonjuro, Gangnam-gu, Seoul, 06273 South Korea

**Keywords:** Rheumatology, Risk factors

## Abstract

Gamma-glutamyl transferase (GGT) is known to promote oxidative stress. As oxidative stress is a key component in the pathogenesis of systemic sclerosis (SSc), we investigated whether GGT levels are associated with the risk of incident SSc. A cohort of individuals without SSc who underwent national health examination in 2009 were extracted from the Korean National Health Insurance Service database. The incidence rate of SSc during the observation period, between 2009 and 2019, was estimated. GGT levels measured in 2009 were categorized into quartiles (Q1 [lowest], Q2, Q3, and Q4 [highest]). Multivariable Cox proportional hazard models were used to estimate the risk of incident SSc according to the quartiles of GGT, using Q1 as the reference. A total of 6,091,788 individuals were included. Incidence rate of SSc was 1.16 per 100,000 person-years over a mean observation period of 9.2 years. After adjusting for age, sex, body mass index, economic income, smoking status, alcohol consumption, physical activity, hypertension, type 2 diabetes, dyslipidemia, and chronic kidney disease, higher quartiles of GGT levels were significantly associated with a higher risk of incident SSc (Q4: adjusted hazard ratio [aHR] 1.807, 95% confidence interval CI 1.446–2.259; Q3: aHR 1.221, 95% CI 0.971–1.536; and Q2: aHR 1.034, 95% CI 0.807–1.324; p for trend < 0.001). Higher GGT levels were associated with a higher risk of incident SSc. These findings could lead to a closer monitoring for high risk individuals and an earlier diagnosis and treatment.

## Introduction

Systemic sclerosis (SSc) is a chronic immune-mediated disease with the highest mortality rates of all rheumatic diseases^1^. Early detection of SSc and timely implementation of treatment are important for better outcomes^2,3^. Identification and close monitoring of individuals who are at a high risk of developing SSc could lead to earlier diagnosis and treatment.

In the pathogenesis of SSc, oxidative stress is well-known to be a key player of vasculopathy, inflammation, autoimmunity, and fibrosis^4^. A recent study has shown that oxidative stress is increased in patients with SSc, and is related to pulmonary hypertension, digital ulcers, and systemic inflammation^5^. Reactive oxygen species (ROS) are involved in endothelial cell activation and apoptosis, and angiogenesis impairment, leading to microvascular damage^4^. ROS also induce M2 macrophage polarization and NLRP3 activation, promoting inflammation in SSc^4,6–9^. With regard to autoimmunity, mice and human data have shown that the oxidation of topoisomerase I results in a break of tolerance to this antigen, leading to the production of anti-topoisomerase I antibodies and increased antigen–antibody reactivity^10,11^. In addition, oxidative stress promotes differentiation of B cells into plasmocytes, leading to increased production of autoantibodies^4,12,13^. Oxidative stress is also involved in T cell activation, favoring differentiation into T helper (Th) 2 and Th17 cells, while limiting differentiation to regulatory T cells^4^. In terms of fibrosis, ROS activate fibroblasts, which in turn increases the activity of ROS^14,15^. Moreover, this vicious cycle applies to the interplay between ROS and transforming growth factor-β, a potent pro-fibrotic cytokine, suggesting that oxidative stress plays an important role in the fibrotic process of SSc^4,16–18^.

Gamma-glutamyl transferase (GGT) is a serologic marker that has been traditionally used for detecting alcohol-related liver diseases and hepatobiliary diseases^19^. More recent studies have shown that enzymatic activity of GGT promotes the generation of ROS^20,21^. Given its pro-oxidant reactions, the implication of GGT in the pathogenesis of several diseases, including cardiovascular diseases, cancers, lung inflammation, and neurological diseases, is being actively researched^20^. However, currently there are no studies evaluating the implication of GGT in SSc. Considering that oxidative stress plays a crucial role in the pathogenesis of SSc, GGT may be a marker associated with the development of SSc.

In this study, we performed a nationwide cohort study and investigated whether the levels of GGT are associated with the risk of SSc development.

## Methods

### Data source and study cohort

The study cohort was extracted from the Korean National Health Insurance Service (NHIS) claims database. The NHIS covers approximately 97% of the total population of South Korea. Data about demographics, and medical- and health-related information on each individual are available in the NHIS database. Data on national health check-ups, which is checked every two years for individuals aged ≥ 40 years or employees of any age, are also available. The national health check-up data include smoking status, alcohol consumption, physical activity, body mass index (BMI), systolic and diastolic blood pressure (BP), and laboratory data such as aspartate aminotransferase (AST), alanine aminotransferase (ALT), and GGT levels^22^.

**Figure 1:**
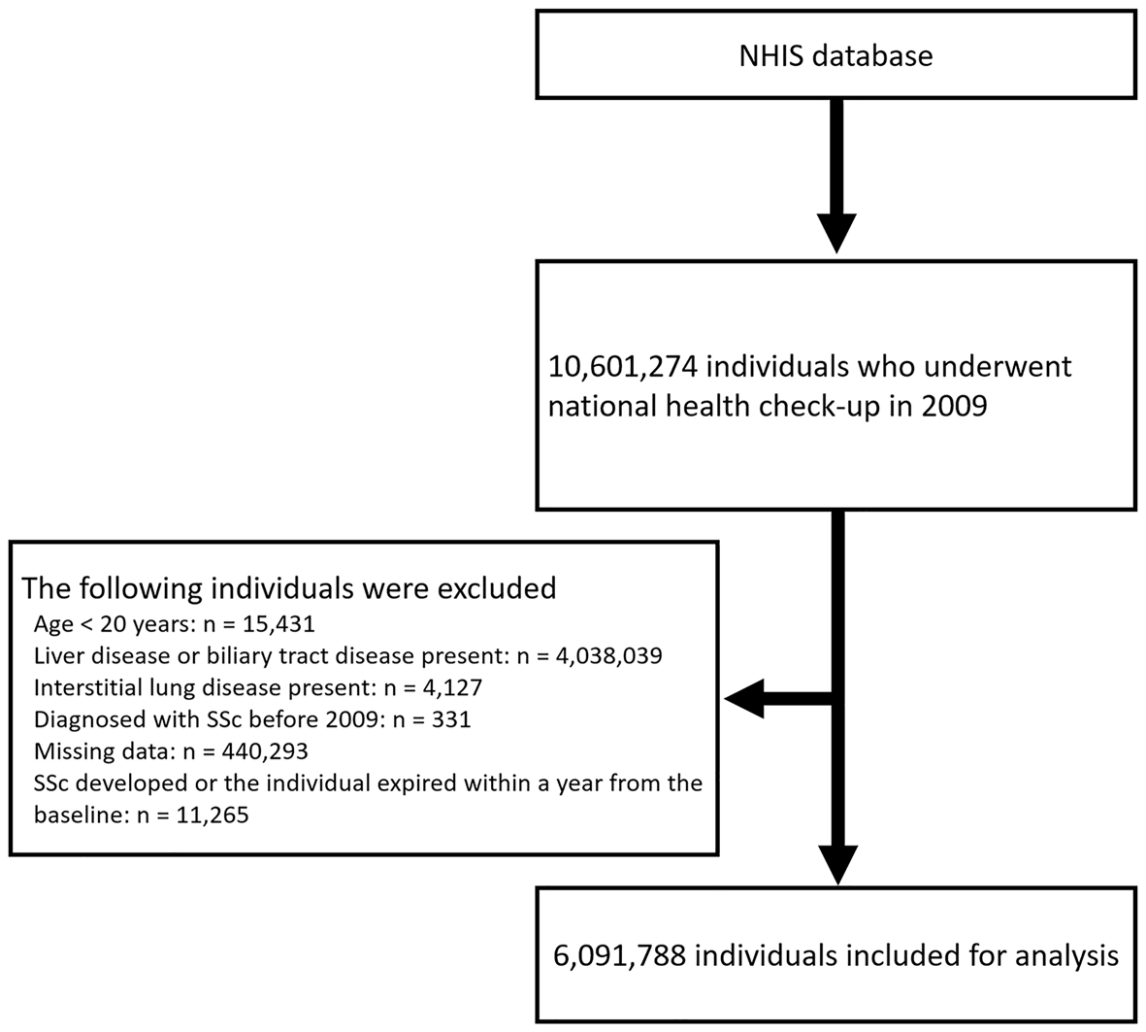
Selection of the study population from the NHIS database. *NHIS* National Health Insurance Service, *SSc* systemic sclerosis.

From the database, individuals who underwent national health check-ups in 2009 were selected (N = 10,601,274). For the selected individuals, the following exclusion criteria were applied: (i) age < 20 years; (ii) had liver disease (International Classification of Diseases-10th Revision [ICD-10] codes K70–K77) or biliary tract disease (ICD-10 codes K80–K87); (iii) had interstitial lung disease (ICD-10 code J84.1); (iv) SSc diagnosis before 2009; (v) data missing; and (vi) SSc developed or the individual died within a year of the health check-up date (Fig. 1). As a result, 6,091,788 individuals were included in the cohort. The observation period was from the date of the health check-up in 2009 (i.e. baseline) to December 2019.

### GGT levels

The GGT levels measured at baseline were categorized into quartiles (Q1, Q2, Q3, and Q4). Q1 was the lowest 25%, and Q4 was the highest 25%. The cut-offs of the quartiles were as follows: Q1 (< 21 IU/L for male, and < 13 IU/L for female), Q2 (< 30 IU/L for male, and < 16 IU/L for female), Q3 (< 49 IU/L for male, and < 22 IU/L for female), and Q4 (≥ 49 IU/L for male, and ≥ 22 IU/L for female).

### Definitions of outcome and covariates

The study outcome was the incidence of SSc, which was defined as a rare intractable disease (RID) with a code of V138^23^. In the Korean RID system, the RID code is given after a thorough review of the fulfillment of the diagnostic criteria provided by the NHIS^22^. Covariates including smoking status, alcohol consumption, and physical activity, were defined based on data from standardized self-reporting questionnaires, which are included in the national health check-up data. According to the questionnaires, smoking status was categorized into non-smoker (who never smoked), ex-smoker (who previously had smoked but not currently), and current smoker (who smokes currently). Alcohol consumption was categorized into non-drinker (0 g/day), mild drinker (> 0 g/day and < 30 g/day), and heavy drinker (≥ 30 g/days). Regarding physical activity, regular exercise was defined as a moderate exercise ≥ 5 days or vigorous exercise ≥ 3 days per week^22^. Other covariates were defined based on ICD-10 codes, prescription, laboratory data, and BP as described in previous studies (Supplemental Table 1)^24^.

### Statistical analysis

Continuous variables that followed normal distribution were expressed as mean ± standard deviation, and continuous variables that followed skewed distribution were Log-transformed and expressed as geometric mean (95% confidence interval [CI]). The normality of the continuous variables was tested using Kolmogorov–Smirnov test and histogram. Categorical variables are expressed as numbers (%). Continuous variables were compared using one-way analysis of variance, and categorical variables were compared using Chi^2^ test. The incidence rate of SSc was calculated as the number of events per 100,000 person-years. Cox proportional hazard analyses were used to investigate the association between baseline GGT levels and incidence of SSc. Using Q1 levels of GGT as the reference, hazard ratios (HRs) and 95% CIs according to the quartiles of GGT levels were estimated. Model 1 was a crude model. Model 2 was adjusted for age and sex. Model 3 was adjusted for age, sex, BMI, income, smoking status, alcohol consumption, and physical activity. Model 4 was additionally adjusted for hypertension, type 2 diabetes, dyslipidemia, and chronic kidney disease (CKD). As a sensitivity analysis, we categorized the levels of GGT to deciles (D1 [lowed 10%] to D10 [highest 10%]) and analyzed the risk of incident SSc according to the deciles of GGT levels, using D1 as the reference. Subgroup analysis was performed to assess whether there is a subset of individuals in whom the association between GGT levels and risk of incident SSc is more pronounced. All *p*-values were two-sided, and a *p*-value < 0.05 was considered statistically significant. Statistical analyses were performed using SAS version 9.4 (SAS Institute, Cary, NC, USA).

### Ethics approval and consent to participate

This study was approved by the Institutional Review Board (IRB) of Gangnam Severance Hospital (No: 3-2022-0338). Owing to the retrospective nature of this study, the requirement for informed consent was waived by the IRB of Gangnam Severance Hospital. This study conformed the ethical guidelines laid out by the 1964 Helsinki declaration.

## Results

### Baseline characteristics

Of the total 6,091,788 individuals included in this study, 654 individuals developed SSc during a mean follow-up of 9.2 ± 1.1 years, accounting for an incidence rate of 1.16 per 100,000 person-years. The comparison of baseline characteristics between individuals with different quartiles of GGT levels is shown in Table 1. Individuals with higher quartiles of GGT levels were older, had a higher BMI, more commonly had a low income (lowest 25%), were more commonly current smokers, heavy alcohol drinkers, less commonly performed regular physical activity, more commonly had hypertension, type 2 diabetes, dyslipidemia, and CKD, had higher systolic BP, diastolic BP, fasting glucose, total cholesterol, low-density lipoprotein cholesterol, triglyceride, AST, and ALT levels, and had lower high-density lipoprotein cholesterol levels and estimated glomerular filtration rates (all p for trend < 0.001). The geometric mean values (95% CI) of GGT in individuals with GGT in Q1, Q2, Q3, and Q4 were 12.75 (12.75–12.76) IU/L, 19.45 (19.44–19.46) IU/L, 26.77 (26.75–26.78) IU/L, and 54.38 (54.33–54.44) IU/L, respectively.

**Table 1:** Comparison of baseline characteristics according to the baseline quartiles of GGT levels.

	Q1 (N = 1,561,626)	Q2 (N = 1,386,761)	Q3 (N = 1,591,037)	Q4 (N = 1,552,364)	P value^a^
Age, years	42.28 ± 14.18	43.86 ± 13.85	45.43 ± 13.55	46.95 ± 12.86	< 0.001
Age groups, years, n (%)					< 0.001
< 40	726,443 (46.52)	569,916 (41.10)	562,479 (35.35)	460,119 (29.64)	
40–64	693,169 (44.39)	685,549 (49.44)	866,965 (54.49)	930,579 (59.95)	
≥ 65	142,014 (9.09)	131,296 (9.47)	161,593 (10.16)	161,666 (10.41)	
Sex, n (%)					< 0.001
Male	834,503 (53.44)	813,543 (58.66)	872,026 (54.81)	847,634 (54.60)	
Female	727,123 (46.56)	573,218 (41.34)	719,011 (45.19)	704,730 (45.40)	
Income, lowest Q1, n (%)	245,284 (15.71)	210,456 (15.18)	246,761 (15.51)	249,542 (16.07)	< 0.001
Smoking, n (%)					< 0.001
Non	1,016,061 (65.06)	808,313 (58.29)	918,443 (57.73)	836,843 (53.91)	
Ex	197,126 (12.62)	199,580 (14.39)	218,404 (13.73)	197,285 (12.71)	
Current	348,439 (22.31)	378,868 (27.32)	454,190 (28.55)	518,236 (33.38)	
Drinking, n (%)					< 0.001
Non	911,359 (58.36)	704,196 (50.78)	756,640 (47.56)	640,028 (41.23)	
Mild	606,979 (38.87)	612,130 (44.14)	713,405 (44.84)	689,540 (44.42)	
Heavy	43,288 (2.77)	70,435 (5.08)	120,992 (7.60)	222,796 (14.35)	
Regular exercise, n (%)	272,996 (17.48)	241,442 (17.41)	268,820 (16.90)	251,428 (16.20)	< 0.001
Hypertension, n (%)	197,894 (12.67)	238,536 (17.20)	360,481 (22.66)	488,378 (31.46)	< 0.001
Type 2 diabetes, n (%)	46,799 (3.00)	56,761 (4.09)	92,395 (5.81)	154,773 (9.97)	< 0.001
Dyslipidemia, n (%)	102,697 (6.58)	144,938 (10.45)	246,605 (15.50)	359,663 (23.17)	< 0.001
CKD, n (%)	88,487 (5.67)	81,829 (5.90)	100,977 (6.35)	103,792 (6.69)	< 0.001
Height, cm	164.81 ± 9.00	165.11 ± 9.02	164.21 ± 9.22	163.66 ± 9.45	< 0.001
Weight, kg	60.66 ± 10.02	62.95 ± 10.99	64.36 ± 11.85	66.57 ± 12.57	< 0.001
BMI, kg/m^2^	22.26 ± 2.68	23.00 ± 2.92	23.75 ± 3.15	24.73 ± 3.38	< 0.001
BMI Level, kg/m^2^, n (%)					< 0.001
< 18.5	97,044 (6.21)	65,828 (4.75)	58,784 (3.69)	35,369 (2.28)	
< 23	886,193 (56.75)	645,388 (46.54)	593,219 (37.29)	431,480 (27.80)	
< 25	341,797 (21.89)	346,289 (24.97)	406,849 (25.57)	381,051 (24.55)	
< 30	224,525 (14.38)	307,063 (22.14)	481,658 (30.27)	604,249 (38.92)	
≥ 30	12,067 (0.77)	22,193 (1.60)	50,527 (3.18)	100,215 (6.46)	
Waist circumference, cm	76.02 ± 7.99	78.34 ± 8.54	80.17 ± 8.98	82.75 ± 9.06	< 0.001
Systolic BP, mmHg	117.92 ± 13.81	120.25 ± 14.21	122.37 ± 14.79	125.91 ± 15.58	< 0.001
Diastolic BP, mmHg	73.42 ± 9.33	75.04 ± 9.60	76.45 ± 9.94	78.78 ± 10.49	< 0.001
Fasting glucose, mg/dL	91.60 ± 15.98	93.41 ± 18.37	95.73 ± 21.30	100.74 ± 27.14	< 0.001
Total Cholesterol, mg/dL	181.88 ± 32.28	190.70 ± 33.45	197.69 ± 35.28	205.95 ± 38.51	< 0.001
HDL-C, mg/dL	57.08 ± 26.88	56.32 ± 26.03	56.16 ± 27.61	56.02 ± 26.56	< 0.001
LDL -C, mg/dL	106.85 ± 35.78	112.73 ± 36.69	116.20 ± 37.88	117.04 ± 40.86	< 0.001
Triglyceride^b^, mg/dL	84.93 (84.87–85.00)	99.17 (99.09–99.26)	114.17 (114.08–114.27)	143.02 (142.89–143.15)	< 0.001
eGFR, mL/min/1.73m^2^	90.07 ± 44.35	88.43 ± 50.27	87.77 ± 49.04	87.4 ± 45.6	< 0.001
AST^b^, IU/L	19.77 (19.76–19.78)	21.14 (21.13–21.15)	22.66 (22.65–22.67)	27.52 (27.5–27.54)	< 0.001
ALT^b^, IU/L	15.25 (15.24–15.26)	17.97 (17.96–17.99)	20.97 (20.95–20.98)	29.09 (29.07–29.12)	< 0.001
GGT^b^, IU/L	12.75 (12.75–12.76)	19.45 (19.44–19.46)	26.77 (26.75–26.78)	54.38 (54.33–54.44)	< 0.001

*ALT* alanine aminotransferase, *AST* aspartate aminotransferase, *BMI* body mass index, *BP* blood pressure, *CKD* chronic kidney disease, *eGFR* estimated glomerular filtration rate, *GGT* gamma-glutamyl transferase, *HDL*-*C* high-density lipoprotein cholesterol, *LDL*-*C* low-density lipoprotein cholesterol.

^a^P for trend.

^b^Geometric mean (95% confidence interval).

### Risk of incident SSc according to the GGT levels

The incidence rate and risk of incident SSc according to the GGT levels are reported in Table 2. The incidence rates of SSc in individuals with GGT levels in Q1, Q2, Q3, and Q4 were 0.95, 0.91, 1.14, and 1.64 per 100,000 person-years, respectively. In the crude model (model 1), individuals with higher quartile levels of GGT had a higher risk of incident SSc than those with Q1 levels of GGT (Q4: unadjusted HR 1.723, 95% CI 1.395–2.127; Q3: unadjusted HR 1.199, 95% CI 0.956–1.503; and Q2: unadjusted HR 0.962, 95% CI 0.752–1.232; p for trend < 0.001). Similar results were observed after adjusting for multiple covariates in model 4 (Q4: adjusted HR 1.807, 95% CI 1.446–2.259; Q3: adjusted HR 1.221, 95% CI 0.971–1.536; and Q2: adjusted HR 1.034, 95% CI 0.807–1.324; p for trend < 0.001).

**Table 2:** Incidence of systemic sclerosis according to quartiles of GGT at baseline.

GGT quartile	Cut-off (IU/L)		N	Event	Duration (pyrs)	IR (/100,000 pyrs)	HR (95% CI)			
	Male	Female					Model 1	Model 2	Model 3	Model 4
Q1	< 21	< 13	1,561,626	137	14,414,650.18	0.95	1 (Ref.)	1 (Ref.)	1 (Ref.)	1 (Ref.)
Q2	< 30	< 16	1,386,761	117	12,793,032.69	0.91	0.962 (0.752–1.232)	0.991 (0.774–1.269)	1.022 (0.798–1.309)	1.034 (0.807–1.324)
Q3	< 49	< 22	1,591,037	167	14,666,014.11	1.14	1.199 (0.956–1.503)	1.109 (0.884–1.392)	1.185 (0.942–1.491)	1.221 (0.971–1.536)
Q4	≥ 49	≥ 22	1,552,364	233	14,243,674.84	1.64	1.723 (1.395–2.127)	1.504 (1.215–1.863)	1.690 (1.353–2.110)	1.807 (1.446–2.259)

Model 1: Crude model.

Model 2: Adjusted for age and sex.

Model 3: Adjusted for age, sex, BMI, income, smoking status, alcohol consumption, and physical activity.

Model 4: Adjusted for age, sex, BMI, income, smoking status, alcohol consumption, physical activity, hypertension, type 2 diabetes, dyslipidemia, and CKD.

P for trend < 0.001 in all models.

*BMI* body mass index, *CI* confidence interval, *CKD* chronic kidney disease, *GGT* gamma-glutamyl transferase, *HR* hazard ratio, *IR* incidence ratio, *pyrs* person-years.

In the sensitivity analysis (Table 3) where GGT levels were categorized into deciles to test the robustness of the association between higher GGT levels and higher risk of incident SSc, similar associations were observed as in the quartile-based analysis.

**Table 3:** Incidence of systemic sclerosis according to deciles of GGT at baseline.

GGT quartile	Cut-off (IU/L)		N	Event	Duration (pyrs)	IR (/100,000 pyrs)	HR (95% CI)			
	Male	Female					Model 1	Model 2	Model 3	Model 4
D1	< 16	< 10	528,683	45	4,872,509.02	0.92	1 (Ref.)	1 (Ref.)	1 (Ref.)	1 (Ref.)
D2	< 19	< 12	623,907	57	5,764,391.68	0.99	1.071 (0.724–1.583)	0.848 (0.573–1.254)	0.864 (0.584–1.278)	0.865 (0.584–1.279)
D3	< 23	< 13	615,410	42	5,675,673.02	0.74	0.801 (0.526–1.220)	0.850 (0.558–1.295)	0.875 (0.574–1.333)	0.880 (0.578–1.341)
D4	< 26	< 15	677,116	74	6,256,303.98	1.18	1.281 (0.884–1.855)	0.916 (0.632–1.328)	0.958 (0.660–1.390)	0.969 (0.668–1.407)
D5	< 30	< 16	503,271	36	4,638,805.16	0.78	0.840 (0.542–1.303)	0.803 (0.518–1.245)	0.852 (0.549–1.322)	0.868 (0.559–1.347)
D6	< 36	< 18	680,492	69	6,275,364.24	1.10	1.191 (0.818–1.733)	0.968 (0.664–1.411)	1.042 (0.713–1.520)	1.068 (0.731–1.559)
D7	< 44	< 21	674,914	81	6,222,038.09	1.30	1.410 (0.979–2.029)	1.055 (0.731–1.521)	1.156 (0.799–1.672)	1.199 (0.829–1.734)
D8	< 56	< 25	585,701	65	5,393,481.89	1.21	1.305 (0.892–1.908)	1.008 (0.688–1.477)	1.129 (0.767–1.661)	1.188 (0.807–1.747)
D9	< 82	< 33	596,950	69	5,490,771.81	1.26	1.361 (0.935–1.981)	1.070 (0.734–1.562)	1.226 (0.835–1.799)	1.310 (0.892–1.923)
D10	≥ 82	≥ 33	605,344	116	5,528,032.92	2.10	2.274 (1.612–3.209)	1.729 (1.222–2.446)	2.024 (1.418–2.891)	2.215 (1.549–3.167)

Model 1: Crude model.

Model 2: Adjusted for age and sex.

Model 3: Adjusted for age, sex, BMI, income, smoking status, alcohol consumption, and physical activity.

Model 4: Adjusted for age, sex, BMI, income, smoking status, alcohol consumption, physical activity, hypertension, type 2 diabetes, dyslipidemia, and CKD.

P for trend < 0.001 in all models.

*BMI* body mass index, *CI* confidence interval, *CKD* chronic kidney disease, *GGT* gamma-glutamyl transferase, *HR* hazard ratio, *IR* incidence ratio, *pyrs* person-years.

### Subgroup analysis

For subgroup analysis, individuals were stratified according to multiple covariates. The results of the subgroup analysis are summarized in Table 4. The association between higher GGT levels (Q4 compared with Q1–Q3) and increased risk of incident SSc was more pronounced in males than in females (adjusted HR 2.483 vs. 1.504, p for interaction = 0.016).

**Table 4:** Subgroup analysis for the association between levels of GGT and incidence of systemic sclerosis.

		GGT	N	Event	Duration (pyrs)	IR (/100,000 pyrs)	HR^a^ (95% CI)	p for interaction
Age groups	< 40 years	Q1-3	1,858,838	59	17,297,621.08	0.34	1 (Ref.)	0.32
		Q4	460,119	24	4,262,848.81	0.56	2.132 (1.321–3.443)	
	40–64 years	Q1-3	2,245,683	319	20,845,675.07	1.53	1 (Ref.)	
		Q4	930,579	187	8,588,562.19	2.18	1.552 (1.287–1.872)	
	≥ 65 years	Q1-3	434,903	43	3,730,400.82	1.15	1 (Ref.)	
		Q4	161,666	22	1,392,263.84	1.58	1.263 (0.753–2.118)	
Sex	Male	Q1-3	2,520,072	72	23,161,255.25	0.31	1 (Ref.)	0.016
		Q4	847,634	51	7,748,364.82	0.66	2.483 (1.712–3.602)	
	Female	Q1-3	2,019,352	349	18,712,441.73	1.87	1 (Ref.)	
		Q4	704,730	182	6,495,310.02	2.80	1.504 (1.248–1.811)	
Income	Q2-4	Q1-3	3,836,923	329	35,423,165.19	0.93	1 (Ref.)	0.40
		Q4	1,302,822	174	11,966,295.15	1.45	1.586 (1.309–1.920)	
	Q1	Q1-3	702,501	92	6,450,531.79	1.43	1 (Ref.)	
		Q4	249,542	59	2,277,379.70	2.59	1.863 (1.338–2.593)	
Obesity^b^	No	Q1-3	3,441,391	340	31,740,138.29	1.07	1 (Ref.)	0.52
		Q4	847,900	155	7,756,998.97	2.00	1.706 (1.404–2.074)	
	Yes	Q1-3	1,098,033	81	10,133,558.68	0.80	1 (Ref.)	
		Q4	704,464	78	6,486,675.88	1.20	1.512 (1.104–2.071)	
Abdominal obesity^c^	No	Q1-3	3,970,256	373	36,664,752.18	1.02	1 (Ref.)	0.68
		Q4	1,114,965	180	10,235,898.59	1.76	1.676 (1.394–2.014)	
	Yes	Q1-3	569,168	48	5,208,944.80	0.92	1 (Ref.)	
		Q4	437,399	53	4,007,776.25	1.32	1.532 (1.033–2.270)	
Current smoking	No	Q1-3	3,357,927	384	31,006,460.05	1.24	1 (Ref.)	0.57
		Q4	1,034,128	203	9,506,456.81	2.14	1.622 (1.358–1.937)	
	Yes	Q1-3	1,181,497	37	10,867,236.92	0.34	1 (Ref.)	
		Q4	518,236	30	4,737,218.04	0.63	1.880 (1.151–3.069)	
Heavy drinking	No	Q1-3	4,304,709	415	39,708,689.17	1.05	1 (Ref.)	0.13
		Q4	1,329,568	215	12,206,540.21	1.76	1.604 (1.350–1.906)	
	Yes	Q1-3	234,715	6	2,165,007.81	0.28	1 (Ref.)	
		Q4	222,796	18	2,037,134.64	0.88	3.322 (1.319–8.369)	
Regular exercise	No	Q1-3	3,756,166	367	34,633,847.98	1.06	1 (Ref.)	0.06
		Q4	1,300,936	191	11,932,543.72	1.60	1.543 (1.285–1.853)	
	Yes	Q1-3	783,258	54	7,239,848.99	0.75	1 (Ref.)	
		Q4	251,428	42	2,311,131.13	1.82	2.361 (1.572–3.545)	
Hypertension	No	Q1-3	3,742,513	341	34,689,624.55	0.98	1 (Ref.)	0.34
		Q4	1,063,986	167	9,822,669.67	1.70	1.724 (1.424–2.088)	
	Yes	Q1-3	796,911	80	7,184,072.42	1.11	1 (Ref.)	
		Q4	488,378	66	4,421,005.17	1.49	1.433 (1.030–1.994)	
Type 2 diabetes	No	Q1-3	4,343,469	404	40,135,318.65	1.01	1 (Ref.)	0.22
		Q4	1,397,591	218	12,857,436.76	1.70	1.688 (1.420–2.005)	
	Yes	Q1-3	195,955	17	1,738,378.32	0.98	1 (Ref.)	
		Q4	154,773	15	1,386,238.08	1.08	1.075 (0.535–2.157)	
Dyslipidemia	No	Q1-3	4,045,184	377	37,350,168.39	1.01	1 (Ref.)	0.83
		Q4	1,192,701	185	10,947,525.32	1.69	1.660 (1.383–1.993)	
	Yes	Q1-3	494,240	44	4,523,528.58	0.97	1 (Ref.)	
		Q4	359,663	48	3,296,149.52	1.46	1.579 (1.047–2.383)	
CKD	No	Q1-3	4,268,131	392	39,418,023.18	0.99	1 (Ref.)	0.66
		Q4	1,448,572	215	13,311,076.41	1.62	1.664 (1.398–1.982)	
	Yes	Q1-3	271,293	29	2,455,673.80	1.18	1 (Ref.)	
		Q4	103,792	18	932,598.44	1.93	1.450 (0.803–2.617)	

*BMI* body mass index, *CI* confidence interval, *CKD* chronic kidney disease, *GGT* gamma-glutamyl transferase, *HR* hazard ratio, *IR* incidence ratio, *pyrs* person-years.

^a^Adjusted for age, sex, BMI, income, smoking status, alcohol consumption, physical activity, hypertension, type 2 diabetes, dyslipidemia, and CKD.

^b^Obesity: No, BMI < 25 kg/m^2^; yes, BMI ≥ 25 kg/m^2^.

^c^Abdominal obesity: No, waist circumference < 90 cm for men and < 85 cm for women; yes, waist circumference ≥ 90 cm for men and ≥ 85 cm for women.

## Discussion

In this large-scale cohort study, we found that higher GGT levels are associated with an increased risk of incident SSc in the general population. This association was consistently observed when GGT levels were categorized in both quartiles and deciles, thus adding robustness to our results. In addition, we found that this association was stronger in males than in females. To our knowledge, this is the first study to assess the association between GGT levels and risk of incident SSc.

Beyond its traditional use as a marker for alcohol-related liver diseases and hepatobiliary diseases^19^, considering its enzymatic role in promoting oxidative stress^20,21^, GGT is now considered a marker for various diseases such as metabolic syndrome, atherosclerosis, arterial plaque, heart failure, diabetes, and several types of cancers^25^. Our data add to growing knowledge that GGT could be considered as a predictive marker of SSc. Considering that GGT promotes oxidative stress^20,21^, and that oxidative stress is an important factor involved in the pathogenesis of SSc^4^, the association between GGT levels and the risk of incident SSc observed in our study is convincing. In addition, independent of its enzymatic activity, GGT upregulates the gene expression of *tissue factor (TF)* in human peripheral blood mononuclear cells, and increases TF-related pro-coagulant activity^26^. As TF-thrombin signaling enhances the fibrotic activity of myofibroblasts in SSc^27^, the upregulation of *TF* expression by GGT may also explain the association between GGT levels and the risk of incident SSc.

In the subgroup analysis, we found that the association between higher GGT levels and increased risk of incident SSc was more pronounced in males than in females. In other words, the association between higher GGT levels and increased risk of incident SSc was weaker in females. As females per se pose a higher risk of incident SSc than males^28^, the influence of GGT on incidence of SSc may be attenuated in females, resulting in a less prominent association between higher GGT levels and increased risk of incident SSc in females.

A number of studies have evaluated the risk factors of incident SSc: middle age (45–64 years) and female sex have been consistently reported as risk factors of incident SSc^28^. In the multivariable models (model 2–4) of our study, age and sex were adjusted and the association between higher quartiles of GGT levels and risk of incident SSc was observed consistently throughout the models. These indicate that GGT levels could be considered as a risk factor of incident SSc, independent of age and sex.

The upper normal limit of GGT level in Korea is 63 IU/L for males, and 35 IU/L for females^29,30^. In our study, the cut-off of Q4 levels of GGT was ≥ 49 IU/L for males, and ≥ 22 IU/L for females, which are lower than the upper normal limits. As the statistically significant association between quartiles of GGT levels and higher risk of incident SSc was observed in the comparison of Q4 vs. Q1 (model 4, adjusted HR 2.038, 95% CI 1.733–2.396) (Table 2), it is important to note that higher levels of GGT are associated with an increased risk of incident SSc, even when the GGT levels are within the upper normal limit. When analyzed in deciles, the cut-offs of D10 levels of GGT were ≥ 82 IU/L for males, and ≥ 33 IU/L for females. That is, the cut-off of D10 levels for males was above the upper normal limit (63 IU/L), and the cut-off of D10 levels for females was approximately the same as the upper normal limit (35 IU/L). The effect size became exponentially larger in the comparison of D10 vs. D1 (model 4, adjusted HR 2.215) compared with the comparison of D9 vs. D1 (model 4, adjusted HR 1.310) (Table 3). Taken together, the risk of incident SSc increases as the GGT level rises within the upper normal limit, and increases overwhelmingly when the GGT level rises above the upper normal limit.

There are few limitations of this study. First, as this was a Korean population-based study, the results of our study may not be generalizable to other ethnic populations. Second, as the data regarding the phenotypes of SSc, such as organ involvement pattern and modified Rodnan skin score, are unavailable in the NHIS database, we could not analyze the association between GGT levels and SSc phenotypes. Third, as this was a retrospective study, causality could not be fully explained. However, we did apply a 1-year lag period for a better causality assessment. Fourth, as this was a nationwide study, interlaboratory variation on GGT levels may exist. However, previous study has reported that the interlaboratory coefficient of variation for GGT is 11.6%, which is an acceptable level^31^.

## Conclusions

**Figure 2:**
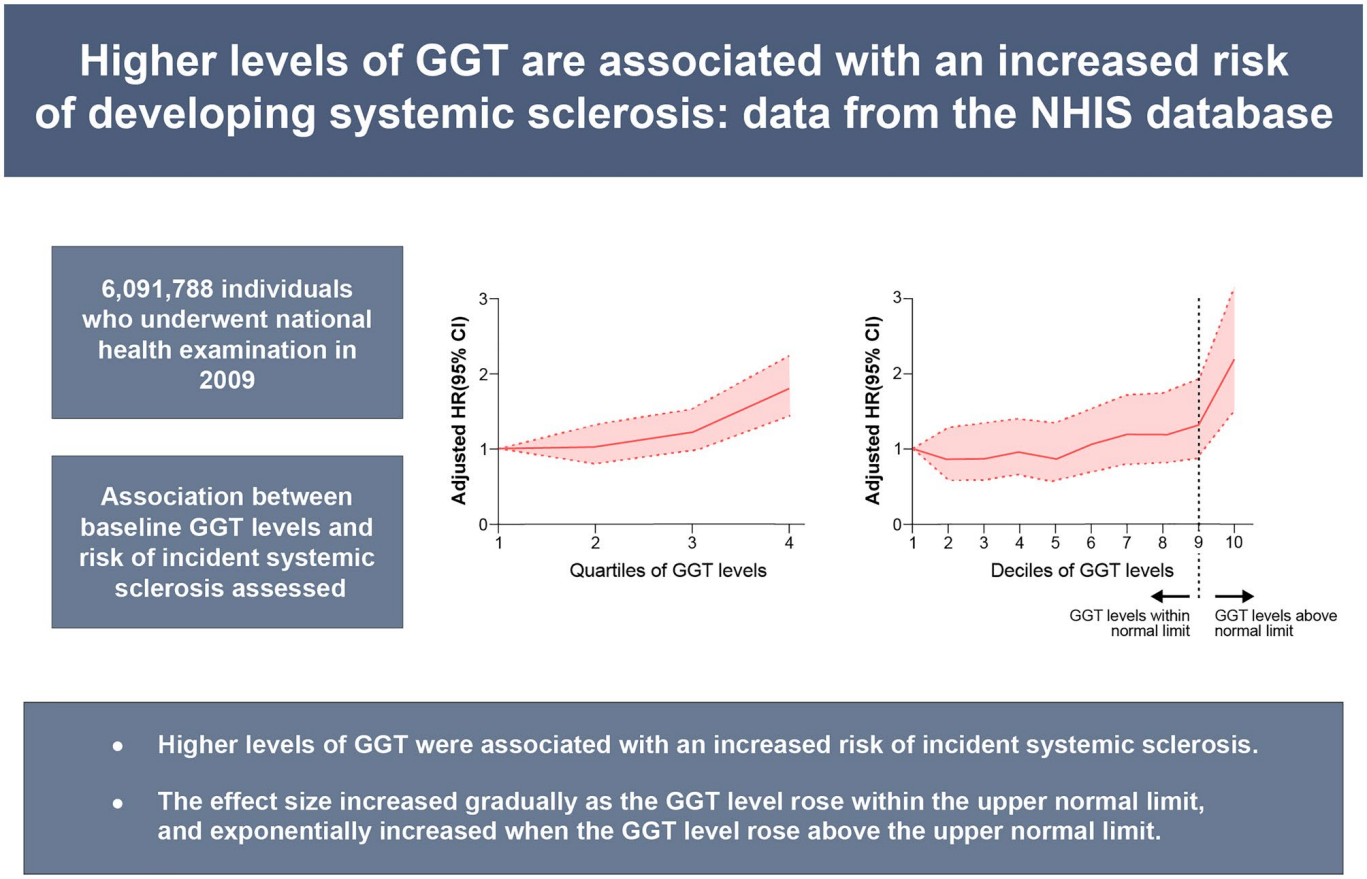
Summary of the present study. *GGT* gamma-glutamyl transferase, *NHIS* National Health Insurance Service, *HR* hazard ratio, *CI* confidence interval.

In conclusion, higher GGT levels were independently associated with an increased risk of incident SSc. The effect size increased gradually as the GGT level rose within the upper normal limit, and exponentially increased when the GGT level rose above the upper normal limit (Fig. 2). Regardless of whether the GGT level was above the upper normal limit or not, the effect size increased significantly as the GGT level rose. Our data suggest that individuals with higher levels of GGT could be considered as having an increased risk of developing SSc. Thus, the clinicians should be aware that close monitoring for the development of SSc is warranted in individuals with higher GGT levels. This could eventually lead to an early diagnosis and treatment of SSc.

### Supplementary Information

Supplementary Table 1.

## Data Availability

All data generated or analyzed during this study are included in this article. All data except the results cannot be shared publicly due to the policy of the national health authorities.

## Abbreviations

SSc: Systemic sclerosis
ROS: Reactive oxygen species
Th: T helper
GGT: Gamma-glutamyl transferase
NHIS: National Health Insurance Service
BMI: Body mass index
BP: Blood pressure
AST: Aspartate aminotransferase
ALT: Alanine aminotransferase
IRB: Institutional review board
RID: Rare intractable disease
ICD-10: International Classification of Diseases-10th Revision
HR: Hazard ratio
CI: Confidence interval
CKD: Chronic kidney disease
TF: Tissue factor
