# Supplementary material for: Higher gamma-glutamyl transferase levels are associated with an increased risk of incident systemic sclerosis: a nationwide population-based study
Source: Sci Rep. 2023 Dec 11;13:21878. doi: 10.1038/s41598-023-49183-1 (PMC10711000; doi:10.1038/s41598-023-49183-1)
Supplement: Supplementary file 1 — Supplementary Table 1. [file 41598_2023_49183_MOESM1_ESM.docx]

Supplemental Table 1. Definitions of covariates

| Covariate | Definition |
| --- | --- |
| Hypertension | (1) ICD-10 codes I10‒I13 and I15 with prescriptions for anti-hypertensive agents  or (2) systolic BP ≥ 140 mmHg or diastolic BP ≥ 90 mmHg |
| Type 2 diabetes | (1) ICD-10 codes E11–14 with prescriptions for anti-diabetic agents  or (2) fasting blood glucose level ≥ 126 mg/dL |
| Hyperlipidemia | (1) ICD-10 code E78 with prescriptions for lipid-lowering agents  or (2) total cholesterol level ≥ 240 mg/dL |
| CKD | eGFR of < 60 mL/min/1.73m^2^ by Modification of Diet in Renal Disease equation |

BP, blood pressure; CKD, chronic kidney disease; eGFR, estimated glomerular filtration rate; ICD, International Classification of Diseases-Tenth Revision.
